# Multimodal Stereotactic Brain Tumor Segmentation Using 3D-Znet

**DOI:** 10.3390/bioengineering10050581

**Published:** 2023-05-11

**Authors:** Mohammad Ashraf Ottom, Hanif Abdul Rahman, Iyad M. Alazzam, Ivo D. Dinov

**Affiliations:** 1 Statistics Online Computational Resource, University of Michigan, Ann Arbor, MI 48104, USA; 2 Department of Information Systems, Yarmouk University, Irbid 21163, Jordan; 3 PAPRSB Institute of Health Sciences, Universiti Brunei Darussalam, Gadong BE1410, Brunei

**Keywords:** deep learning, 3D tumor segmentation, encoder–decoder, Znet, multimodal neuroimaging data

## Abstract

Stereotactic brain tumor segmentation based on 3D neuroimaging data is a challenging task due to the complexity of the brain architecture, extreme heterogeneity of tumor malformations, and the extreme variability of intensity signal and noise distributions. Early tumor diagnosis can help medical professionals to select optimal medical treatment plans that can potentially save lives. Artificial intelligence (AI) has previously been used for automated tumor diagnostics and segmentation models. However, the model development, validation, and reproducibility processes are challenging. Often, cumulative efforts are required to produce a fully automated and reliable computer-aided diagnostic system for tumor segmentation. This study proposes an enhanced deep neural network approach, the 3D-Znet model, based on the variational autoencoder–autodecoder Znet method, for segmenting 3D MR (magnetic resonance) volumes. The 3D-Znet artificial neural network architecture relies on fully dense connections to enable the reuse of features on multiple levels to improve model performance. It consists of four encoders and four decoders along with the initial input and the final output blocks. Encoder–decoder blocks in the network include double convolutional 3D layers, 3D batch normalization, and an activation function. These are followed by size normalization between inputs and outputs and network concatenation across the encoding and decoding branches. The proposed deep convolutional neural network model was trained and validated using a multimodal stereotactic neuroimaging dataset (BraTS2020) that includes multimodal tumor masks. Evaluation of the pretrained model resulted in the following dice coefficient scores: Whole Tumor (WT) = 0.91, Tumor Core (TC) = 0.85, and Enhanced Tumor (ET) = 0.86. The performance of the proposed 3D-Znet method is comparable to other state-of-the-art methods. Our protocol demonstrates the importance of data augmentation to avoid overfitting and enhance model performance.

## 1. Introduction

Contemporary deep learning techniques are widely used in many fields such as agriculture, self-driving cars, fraud detection, and healthcare [1,2,3,4]. Adequate attention to deep learning applications in healthcare emerged recently, including brain tumor diagnostics, detection, and segmentation [5,6,7]. Massive amounts of valuable, multi-source, spatiotemporal, and multiscale data have recently become available in many applied, theoretical, experimental, data science, and healthcare domains [8]. Early detection, accurate prognostication, and precise tracking of diseases contribute heavily to saving lives, finding optimal treatments, and reducing the economic burden for patients and healthcare systems. Similar benefits of machine learning and artificial intelligence are applied to studies of brain tumors and neuro-oncology [9]. In this work, we will present a deep convolutional neural network (CNN) approach, 3D-Znet, to learn the neuroimaging affinities and segment prospective brain tumors using publicly available datasets, BraTS (2020). The 3D-Znet approach is originally inspired by the variational encoder–decoder framework and the skip connection concept to enable the model to reuse features on multiple levels. The model was evaluated using the BraTS (2020) datasets. Assessment of the proposed approach indicates high overall mean dice coefficient scores for whole tumor (0.91), tumor core (0.85), and enhanced tumor (0.86) masks. The augmentation of the original data sample and appropriate data preprocessing provided a performance boost and enhanced the model predictions.

**Figure 1:**
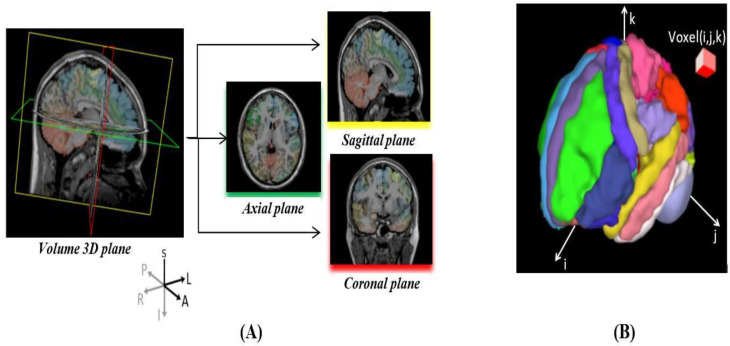
Part (**A**) is the anatomical coordinate system (ACS); the sagittal plane is vertical to the ground, traversing from right (R) to left (L). A coronal plane is vertical to the ground, perpendicular to the sagittal plane and spanning from anterior (A) to the posterior (P) part of the brain. The transverse axial plane is horizontal, traversing from superior (S) to the inferior (I) part. Part (**B**) is the voxel coordinate system with *i*, *j*, and *k* coordinates of a point [15,16].

**Figure 2:**
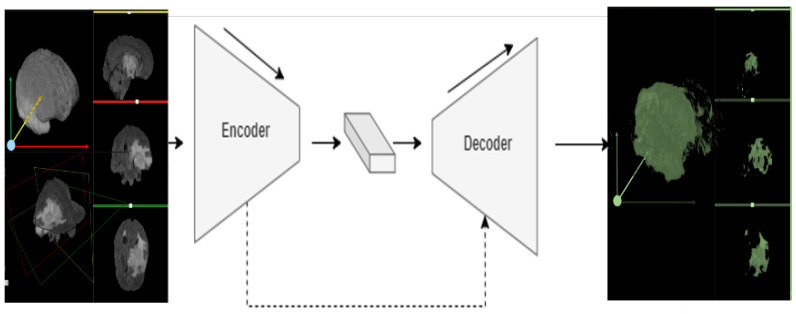
A schematic of an encoder–decoder architecture that maps inputs to outputs preserving the dimensions of the imaging input and the semantic segmentation output. Skip connections are used in some architectures to transfer feature representation from encoder to decoder such as in the Unet [20] and Vnet [21] CNN models.

High-resolution stereotactic medical imaging is critical for patients with brain tumors. Such non-invasive data acquisition protocols rapidly evolve over time, and this drives the development of powerful AI techniques that transform these 3D imaging scans into actionable information and knowledge that can improve the care of patients. Working with medical images such as computed tomography (CT) and magnetic resonance imaging (MRI) is challenging due to data complexity, the large size of the data, and the variability of coordinate representation systems and storage formats [10]. In practice, it is essential to understand the data and medical coordinate systems before applying deep learning techniques, e.g., anatomical and voxel (3D volume-element corresponding to 2D pixel elements) coordinate frameworks. Figure 1A shows the anatomical coordinate system (ACS). ACS comprises three cardinal projection planes illustrating the basic anatomical position of organs in the human body as objects in a solid dense 3D scene. The planes describe the MRI volume orientation. Sagittal cross-sections show 2D image projections of the volume from a side angle (traversing left-to-right direction), the coronal plane from the front view (traversing back-to-front), and the axial plane reveals images as taken from the top down (transverse traversing from top-to-bottom), and Figure 1B shows the brain voxel coordinate system [11,12,13,14]. Deep learning systems provide thriving environments for computer vision and semantic segmentation applications. Variational autoencoder–autodecoders are deep learning networks commonly used to translate an input *x* into an output *r* (*x*) via a two-step process—encoder and decoder. Figure 2 shows a demonstration of an autoencoder

DNN framework. In the encoding phase, the network takes an input *x* and lowers the representation (size) of *x* as it moves through the network layers. This process continues until a certain bottleneck is reached, where the output represents a small feature representation. The subsequent decoder phase does the opposite inflationary process by increasing the feature representation to produce an output *r* (*x*) with the same dimension as the initial network input *x* [17,18]. In principle, depending on the application, the output layer may generate objects of size either smaller or larger than the size of the initial input. For example, for producing a higher resolution image reconstruction (super-resolution) from input size 32 × 32, an additional layer can be added at the end of the network so that the decoder outputs a larger image of size 64 × 64. However, for semantic segmentation, the output size is usually equal to the input size [19].

**Figure 3:**
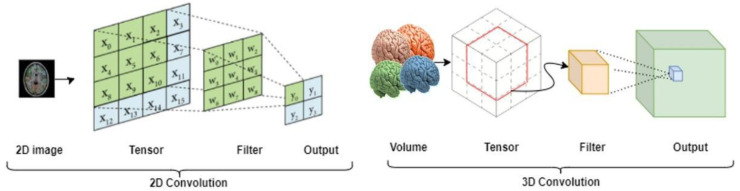
Schematics of 2D and 3D convolution layers; 2D convolution is suitable for classical pixel images, such as CT scans, whereas 3D convolution is used for stereotactic volumetric data, such CT and MR images [22,23,24].

Deep convolutional neural networks (DCNN) are neural networks based on artificial neurons that are structured into layers. The network layers are connected using virtual edges carrying model weights that are computationally estimated during the DCNN model fitting. The initial layer is called the input layer, and the final layer is the output layer. Intermediate hidden layers, located between input and output layers, recursively transform the feature space of one layer to the next. CNNs contain convolutional layers and non-convolutional layers. Convolutional layers include a kernel (filter) to extract features from the preceding input. Patterns are iteratively learned by sliding the filter over the preceding input and calculating the dot-product of the filter and the prior input; a process called (kernel) convolution, and the result of convolving is called a feature map. In the early stages of the CNN fitting, feature maps contain basic patterns, such as edges and corners (basic building blocks). In contrast, later feature maps deeper into the network layers expose more refined patterns (details) that contribute to forming the final output. Among a series of convolutional layers, the feature maps down-sample the images using pooling operations. CNNs are adaptive and can be used to obtain solutions for various types of data in multiple dimensions. Specifically, 2D convolution is used for 2D imaging data indexed in the height and width dimensions and representing scalar or vector intensities, such as gray-scale or RGB images. Higher-dimensional (hyper) volumes, such as 3D solids, require 3D and appropriate higher-dimensional convolutions that are suitable for data of the given dimension (e.g., height, width, and depth for 3D volumes such as MR images). Figure 3 shows the architectural difference between 2D and 3D convolutions [22,23,24].

The study introduces a new approach for segmenting 3D MR volumes using a deep neural network called the 3D-Znet model. This model is an improvement of the variational autoencoder–autodecoder Znet method and uses fully dense connections to improve its performance. The architecture includes four encoders and four decoders, each containing double convolutional 3D layers, 3D batch normalization, and an activation function. The model was trained and validated using the BraTS2020 dataset, which contains multimodal tumor masks. The evaluation of the model showed that it performed well with dice coefficient scores of 0.91 for Whole Tumor, 0.85 for Tumor Core, and 0.86 for Enhanced Tumor, which are comparable to other state-of-the-art methods. The study also emphasizes the importance of data augmentation in enhancing the model’s performance and avoiding overfitting. The rest of the paper is organized as follows: First, we give an overview of the most related work in the field of medical images segmentation, secondly, we describe the methodology, preprocessing phase, the architecture of the proposed model, the training phase, and the evaluation methods. Thirdly, we discuss the experimental results and a comparison with previous results in the literature, and finally is the conclusion and future work.

## 2. Related Work

In their study [25], Karayegen and Aksahin proposed a convolutional neural network approach to diagnose and segment 3D brain tumors using a deep neural network pretrained on the 2020 Brain Tumor Segmentation (BraTS2020) dataset [26]. The authors had normalised the dataset into two classes (background and tumor), however, the dataset has four class categories. During data preprocessing, the authors used histogram equalization to enhance the classifications of edges in each region. To solve memory issues and enhance training performance, they used a random patches mechanism (80 and 90 patches) of sizes 36 × 36 × 155 and 40 × 40 × 155. The evaluation results showed an ability to diagnose and segment tumors with promising results.

Another study by [27] attempts 3D brain tumor segmentation using SegNet algorithm on BraTS dataset. In this study, the investigators trained all the modules separately and integrated them during the post-processing. The four feature maps fused to form one feature map and then a decision tree algorithm was used to classify the output into malignant and benign. Results and evaluation showed a potential for this approach for brain tumor segmentation. A recent report [28] proposed a fusion deep learning called RMU-Net model for 3D semantic segmentation of BraTS datasets. The model is motivated by U-net and MobileNetV2. RMU-Net’s training time is higher than other well known segmentation models, however, the model produces promising results. Another recent study [29] proposed the cascaded V-Nets approach for brain tumor segmentation for multimodal brain MR imaging. V-Net is considered as a well-performing approach in semantic segmentation using a cascaded structure and ensemble method to enhance segmentation results. The model architecture consists of encoder, decoder, and skip connections. The approach also suggests segmenting the whole tumor first and then splitting the output into edema, enhancing tumor, and necrosis. The architecture was trained on the BraTS data and validated independently using local hospitals datasets showing considerable improvements in quality of tumor segmentation. The approach of an independent previous study [29] relied on the usage of prior knowledge, training the model jointly on 3D and 2D data, using ensembling methodology, and introducing post-processing to gain better tumor segmentation. The authors utilized three UNets with distinct inputs, then ensembled the equivalent three outputs, and finally applied the post-processing techniques. The first Unet network used 3D patches of multimodal MR images, the second UNet employed brain parcellation as an extra input, and the last network used 2D slices of multimodal MR images. Then brain parcellation and probability maps for each class from the prior network were obtained and tested using BraTS (2018), BraTS (2020), and other local datasets. The final results for this approach were promising, however, compared to other methods, training time is substantially increased due to using multiple Unet DCNNs. A recent study [30] proposed a brain tumor segmentation method based on an ensemble of 3D U-Nets with different hyper-parameters trained on non-uniformly extracted patches. They created a brain tumor segmentation method using an ensemble of 3D U-Nets. Six networks with varying numbers of encoding/decoding blocks, patch sizes, and loss weights were trained and ensembled by averaging the final prediction probabilities. The ensemble model outperformed any of the single models in terms of results. However, the ensemble method requires extensive computational power and is time-consuming. Moreover, [31] proposed a novel transformer-based method for 3D medical image segmentation. The method is effective at extracting local and global characteristics; in addition, the authors designed a combination of transformer structure and CNN, as well as an ETrans (Enhanced Transformer) model, to enhance detail feature extraction. This model was used to extract local detailed features, allowing the model to perform well in segmenting categories that occupy a small portion of the image. However, due to the extensive use of the transformer structure, the performance when segmenting the edges was insufficient. Another study [32] used magnetic resonance images (MRI) to classify images of Alzheimer’s disease (AD) using deep convolutional neural networks (CNN) involving CNN and transfer learning (Visual Geometry Group (VGG)16 and VGG19). Images of Alzheimer’s disease were divided into four categories by neurologists, and the results were assessed using a range of metrics, where VGG-19 was the best in three categories. The research in [33] proposed a machine-learning diagnostic system for COVID-19. For a quicker and more accurate detection of possible COVID-19 instances, four machine learning algorithms—Random Forest (RF), XGBoost, and Light Gradient Boosting Machine (LGBM)—were applied. The dataset utilized the pertinent symptoms for the identification of a suspicious person from COVID-19 symptoms. The results showed that real-time data capturing can efficiently diagnose COVID-19 patients. A recent study [34] suggested a three-stage approach to address brain tumor segmentation. First, a morphological operation pre-processing is applied to remove the skull bone from the image. Then, the particle swarm optimization (PSO) algorithm with a two-way fixed-effects analysis of variance (ANOVA)-based fitness function is utilized to find the optimal block containing the brain lesion. Finally, the K-means clustering algorithm is used to distinguish the detected block as tumor or non-tumor. The study used the BraTS 2015 database and their private dataset from Kouba imaging center-Algiers (KICA), which showed the model’s capability to segment brain tumors.

Despite the availability of various methods and algorithms for brain tumor segmentation, achieving high accuracy in detecting the tumor area and distinguishing it from healthy brain tissue remains challenging and requires further investigation, and the development of methods that are more efficient and effective in detecting and segmenting brain tumors will contribute to the field, especially in cases where the tumor size is small or the tumor is located in a complex area of the brain.

## 3. Methods

### 3.1. Dataset and Pre-Processing

In our study, we used the BraTS 2020 dataset to evaluate the performance of the 3D-Znet model [34,35]. The dataset consists of multi-modal MR images for 369 patients that were captured using two methods of segmentation consisting of Gross total resection (*n* = 359) and Subtotal resection (*n* = 10). Two types of brain tumor neuroimaging data were available, which consisted of high-grade gliomas (*n* = 237) and low-grade gliomas (*n* = 132). The meta-data provides two characteristics of the patients from whom the scans were extracted—the survival (days) and age (years). Using linear regression analysis [2], it was observed that there was a significant downward relationship between length of survival and age. A 10-year increase in age decreased the length of survival by 118 days (*b* = −118; 95% CI: [−160, −70]; *p* < 0.001). These results reflect the importance of this study to develop a better detection tool for early detection to improve the survival rate of patients with brain tumors.

**Figure 4:**
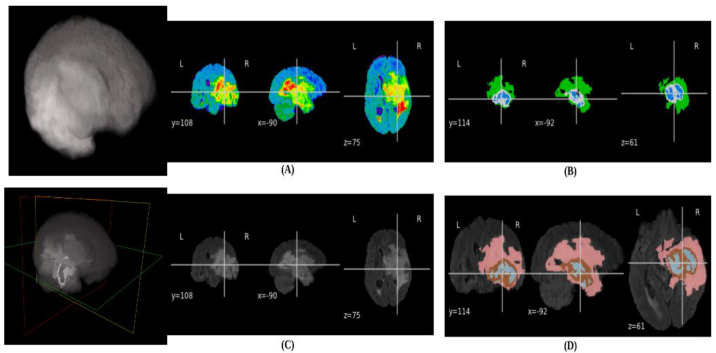
(**A**) Cardinal projection cross sections of the Echo Planar Imaging (EPI) data: Coronal, Axial, and Sagittal planes of a sample Flair volume. (**B**) EPI plots: Coronal, Axial, and Sagittal for a ground truth volume (mask). (**C**) Anatomical plots of Coronal, Axial, and Sagittal for a sample Flair volume. (**D**) Region of Interest overlap between ground truth and Flair volumes: Coronal, Axial, and Sagittal cross sectional planes.

The data (multimodal 3D Brats 2020 scans) were compiled from 19 contributing sites [35] and are available in compressed neuroimaging NIFTI file format (.nii.gz) [36]. For each patient, there are five volumes, and each volume dimension is 240 × 240 × 155 representing height, width, and depth, respectively: fluid-attenuated inversion recovery (Flair), T1-weighted (T1), contrast T1-weighted (T1ce), T2-weighted (T2), and ground truth segmentation (seg). The ground truth volume (seg) has four distinct pixel values: no tumor (the value of 0), non-enhancing tumor core NET (the value of 1), peritumoral edema ED (the value of 2), enhancing tumor ET (the value of 4), and the value of 3 represents missing label. The ground truth was segmented manually by one-to-four expert neuro-radiologists [26,36,37]. Figure 4 shows the sample data volume from the BraTs dataset using Nilearn (Statistics for NeuroImaging in python) [38]. The raw or compressed NIFTI files can also be easily displayed using the SOCR BrainViewer webapp (https://socr.umich.edu/HTML5/BrainViewer/ (accessed on 6 March 2023).

The brain MR images were collected from 19 institutions. This site heterogeneity requires pre-processing to establish corresponding homologies between datasets from different locations and enhance model performance. The 3D volumes were co-registered to a standard anatomical template (an atlas) of an exact resolution of 1 × 1 × 1 mm^3^, and then skull-stripped [34] to remove extra-cerebral tissue. Min-max normalization was used to temper intensity variation and to scale the intensity to a uniform scale between 0 and 1. We also reshaped the original volumes dimension from 240 × 240 × 155 to 128 × 128 × 128, which represent the stereotactic height, width, and depth (slices) dimensions, respectively. Resizing can affect model accuracy; however, it was performed to meet the available hardware resources and to minimize training time.

**Figure 5:**
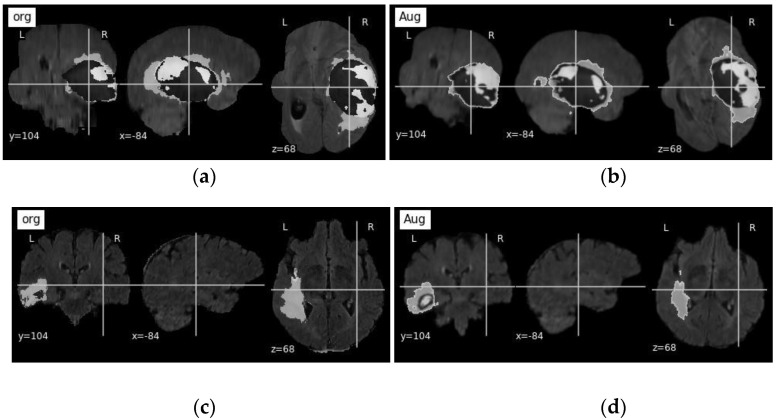
Sample of data augmentation using rotation technique of 10 and −5 degrees: (**a**) original volume; (**b**) augmented volume rotated by 10 degrees; (**c**) original volume; and (**d**) augmented volume rotated by −5 degrees.

**Figure 6:**
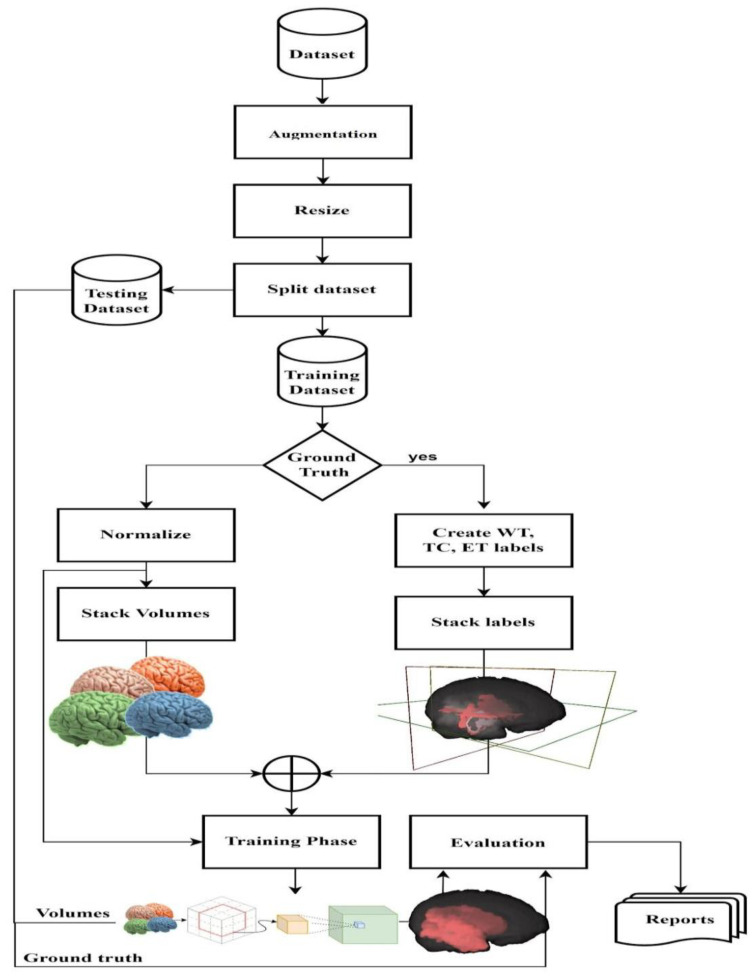
Flowchart of the proposed end-to-end Znet pipeline workflow protocol.

Data augmentation is used to produce more samples of data from the available dataset using techniques to modify the existing data, such as flipping images, zooming in and out, rotating by a certain angle, or using more complex synthetic algorithms. We used rotation by angles −5° to 15° degrees to expand the training data from 369 to 1845 volumes, where 60% were used for training and the rest for testing. Figure 5 shows a sample volume before and after data augmentation. The segmented volume (ground truth) was used to generate three volumes: Whole Tumor (WT), Enhancing Tumor (ET), and Tumor Core (TC). WT volume is a copy of ground truth volume, where pixel values are a combination of nonenhancing tumor core NET (1), peritumoral edema ED (2), enhancing tumor ET (4), and the values of the remaining pixels are non-tumor (value 0). ET is another copy of the segmented volume, where pixel values correspond to enhancing tumor ET (4) and the rest of the pixel values correspond to non-tumor (value 0). The third copy of the segmented volume (TC) has the values for non-enhancing tumor core NET (1), enhancing tumor ET (4) and, the rest are 0 (non-tumor). Finally, the three generated volumes are stacked together to form one multi-modal tensor of dimension (3 × 128 × 128 × 128). On the other hand, actual image volumes fluid-attenuated inversion recovery (Flair), T1-weighted (T1), contrast T1-weighted (T1ce), and T2-weighted (T2) are stacked together to form one multi-modal volume with the dimension of (4 × 128 × 128 × 128). Figure 6 shows the entire workflow pipeline demonstrating the data preprocessing, model fitting, Znet assessment, and result reporting protocol.

### 3.2. 3D-Znet Architecture

The prior 2D-Znet model encoder–decoder framework [39] inspired the new 3D-Znet architecture, which is used for stereotactic (3D) neuroimaging volumes, such as multimodal MR images. The 3D-Znet architecture relies on fully connected connections (dense connections), which is very powerful in biomedical applications, segmentation, and prediction [40].

**Figure 7:**
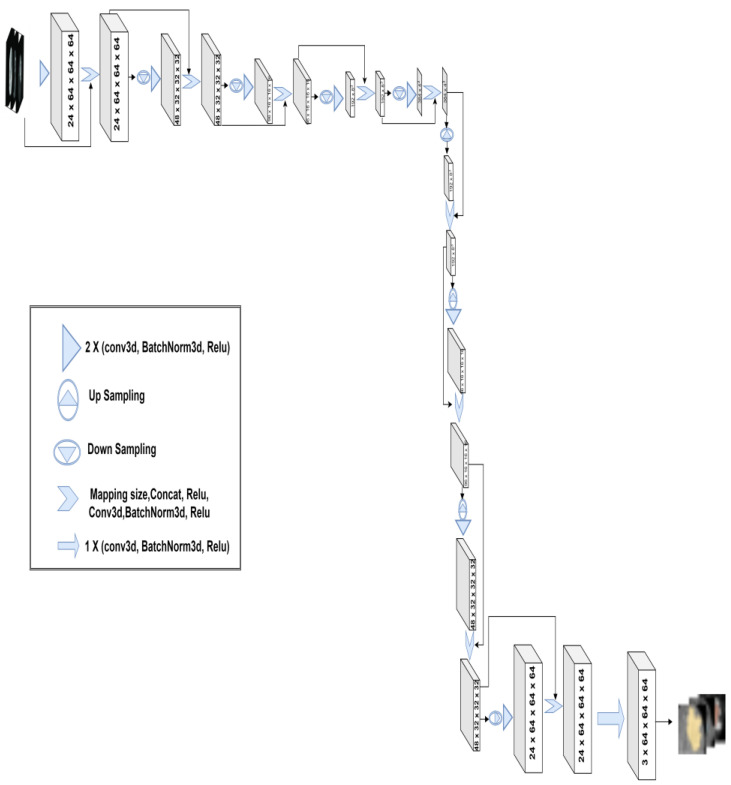
The proposed 3D-Znet architecture for 3D MRI brain tumor segmentation, composed of encoder–decoder blocks and fully connected connections (dense connections) for a sample spatial dimensions of (3,64,64,64).

The objective of dense connections is to enable the model to reuse features on multiple levels to improve model performance [41], where every block of input layers is densely connected to the subsequent block of nodes in the next layer. 3D-Znet incorporates four encoders and four decoders paired with input and output blocks. Each block of the encoder–decoder consists of double convolutional 3D layers, 3D batch normalization, and an activation function (ReLU). These are followed by size normalization between inputs and outputs to facilitate network concatenation and to produce the inputs for the subsequent encoder–decoder blocks. Encoder blocks differ from decoder blocks by using the 3D-maxpooling to downsample the input along its width, height, and depth. In contrast, the decoder block utilizes upsampling to generate super-resolution inputs and retain the original volumetric dimensions at the last decoder block. Conv3d uses a 3D convolution over an input tensor. In the simplest case, the output value of the conv3d with input size (N, Cin, D, H, W) is a tensor sized (N, Cout, Dout, Hout, Wout), where N is the batch size, C is the number of channels, D is the depth, H, and W are highest and width, respectively. The conv3d parameters are kernel_size = 3, stride = 1, and padding = 1. The role of batch normalization is to make the training quicker and more stable by re-centering and re-scaling the input tensor. The overall 3D-Znet architecture is illustrated in Figure 7.

### 3.3. Evaluation Metrics

A key part of DNN model evaluation requires reliable similarity measures to quantify the similarity (or discrepancy) between the ground truth output and the DNN-generated output (Znet segmented masks). Assessing image segmentation is non-trivial, since there is no unique and perfect evaluation framework [42,43]. However, metrics such as the dice similarity coefficient are useful for evaluating and tracking the similarity between segmented image outputs and the corresponding target tumor masks [28]. The set-theoretic *dice* coefficient is a measure comparing a pair of sets, *MS* (machine segmentation) and *GT* (ground truth), by calculating their intersection sizes divided by their union. The analytical form for the *dice* coefficient is shown in following equation:

$$dice=\frac{\left|MS\cup GT\right|}{\left|MS\cap GT\right|}$$

### 3.4. Model Training

We trained the model for 50 epochs using an adaptive moment estimation (ADAM) optimizer [44], images volumetric of size 128 × 128 × 128 pixels, a batch size of 1 due to memory limitation, and a binary cross-entropy loss function [45]. Hardware and software specifications include 2 × 16-core Intel Xeon CPUs, 1× NVidia Titan 12 GB GPU, 128 GB RAM, 6 TB HDD storage, Ubuntu 18.04.5 LTS, Nvidia GPU driver v460.91, CUDA 11.2 + CuDNN 8.1, Torch v1.10.0, torchvision v0.11.1, Spyder v4.2.5, and other supporting python libraries, with a running time of about 45 min for each epochs. See the project GitHub repository for details, code, and complete end-to-end protocol, https://github.com/SOCR/DL_ZNet_3D_BrainSeg (accessed on 6 March 2023).

## 4. Experimental Results

In this section, we summarize the 3D-Znet model performance using a series of experiments aiming to identify an optimal multimodal tumor mask segmentation using the BraTS (2020) public dataset containing the stereotactic MR volumes. Initially, the data augmentation process was necessary to expand the data samples from the original sample of 369 raw volumes to a larger 1, 845 sample of training volumetric data using 3D affine transformations. The resulting augmented dataset was divided into training (80%) and testing (20%) sets. Then, all stereotactic data were resized to 128 × 128 × 128 tensors to reduce training complexity and fit the joint 3D-Znet model fitting on all training data within the available RAM limits. Each data sample contains the annotated mask (ground truth), which was processed to obtain three masks called ET (Enhanced Tumor), TC (Tumor Core), and WT (Whole Tumor). The results of training the previously discussed approach 3D-Znet (Figure 7) showed a mean dice correlation of 0.91 for segmenting the whole tumor, 0.85 for tumor core, and 0.86 for segmenting the most difficult enhanced tumor. This corresponds to an overall average dice segmentation coefficient of 0.87. These results provide strong evidence of the ability of the proposed 3D-Znet DCNN method to reliably segment different types of brain tumors where the DCNN-generated tumor masks are in very good agreement with human expert delineations and other state-of-the-art models (see Table 1).

**Figure 8:**
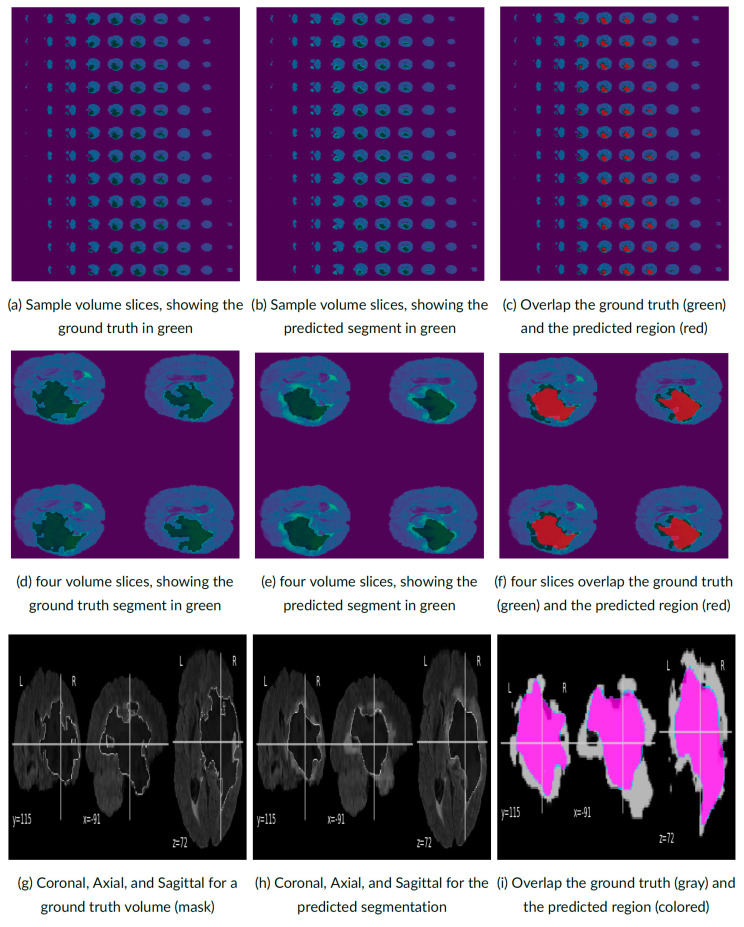
An example of applying the 3D-Znet model to segment the expected brain tumor mask using one random validation-set test-case.

Some visualization examples of the Znet output on testing data are shown in Figure 8. To visually inspect the raw brain images, the corresponding anatomical expert-drawn manually delineated tumor masks, and contrast these against the 3D-Znet predictions, the figure shows axial 2D cross-sections of the 3D volumes. The left panels show the ground truth tumor masks superimposed on the observed MRI volumetric data (image sections) and displayed in green color. The middle panels depict the 3D-Znet prediction masks overlayed on the MRI sections, and the right panels illustrate the overlap between ground truth and DNN-derived tumor masks. There is good agreement between the actual (human expert) and the 3D-Znet (CNN) predicted tumor boundaries. The latter appear a little more regular, smoother, and less complex compared to the native masks, which tend to have highly curved boundaries. Subsequent studies may need to investigate the issue of DCNN regularization of predicted imaging results, understand the underlying causes, and potentially correct for or adjust the DCNN parameters to allow for more irregular boundary shapes.

## 5. Conclusions

In this manuscript, we proposed an efficient deep convolutional neural network (CNN) approach, 3D-Znet, to learn the stereotactic neuroimaging affinities and segment prospective brain tumors using publicly available datasets, such as BraTS (2020). The same 3D-Znet model can be retrained, or refined using transfer learning, on other supervised learning problems. The proposed approach was originally inspired by the variational encoder–decoder framework and the skip connection concept to enable the model to reuse features on multiple levels. The 3D-Znet model includes four encoders and four decoders along with input and output blocks. The model was evaluated using the BraTS (2020) datasets. Assessment of the proposed approach indicates high overall mean dice coefficient scores for whole tumor (0.91), tumor core (0.85), and enhanced tumor (0.86) masks. The augmentation of the original data sample and appropriate data preprocessing provided a performance boost and enhanced the Znet model predictions. In addition, we found that data augmentation plays an important role in avoiding model overfitting. On the other hand, data augmentation requires significantly more computational resources, longer training time, and significant computational infrastructure during the learning process. These upfront costs of training the Znet model on augmented data do not present a computational burden during the subsequent Znet tumor prediction, model validation, translations, and clinical assessment. We found that predicting the enhanced and tumor concentrated masks represented the most challenging tumor segmentation problem in the Brats (2020) archive. This may be explained by the low number of pixels in these types of tumors and potentially highly-subjective diagnoses by trained neuro-radiologists for complex tumor types. Prospective work to expand, improve, and generalize the proposed Znet model may involve alternative strategies to overcome limited data samples, swapping the 3D convolutional layers in the DCNN by 3D wavelet or 3D fractal encoding-decoding transformations, and utilizing different learning techniques. The problem of generating efficient, reliable, and realistic algorithms for segmenting high-dimensional and multimodal neuroimaging data with supervised ground truth labels is difficult. Solutions to this problem may have direct implications to advancing clinical care as well as provide novel mechanisms to synthetically generate unlimited (simulated) realistic neuroimaging data that can be used to train the next-generation AI/ML algorithms that are more sensitive, expeditious, and pragmatic. In addition, transfer learning approaches based on the proposed 3D-Znet may also reduce the training time and provide more accurate predictions. Deep learning models are complex models that often have a large number of parameters. This complexity can lead to inherent uncertainty, which refers to the fact that the model may not fully capture the underlying data distribution. Moreover, there are many hyperparameters that can be tuned in deep learning models, such as learning rate, batch size, and dropout rate. The choice of hyperparameters can affect the model’s performance and uncertainty. Addressing these uncertainties is an ongoing area of research in deep learning.

All software, pretrained 3D-Znet models, and end-to-end electronic python notebook used in this study are available in the project GitHub repository (https://github.com/SOCR/DL_ZNet_3D_BrainSeg, accessed on 6 March 2023).

## Figures and Tables

**Table 1:** Comparison of the 3D-Znet model to previous segmentation models based on the mean dice coefficient using three types of outputs; tumor core (TC), enhanced tumor (ET), and whole tumor (WT).

Model Information	Dice Coefficient				Dataset	Ref.
	WT	TC	ET	Avg.		
Robust Deep Learning and Ranger for brain tumor segmentation 3D Unet	88.9%	81.4%	84.1%	85.0%	Brats2020	[46]
Modality-Pairing learning method using 3D U-Net	89.1%	81.6%	84.2%	84.9%	BraTS2020	[47]
Hybrid High-resolution and Non-local Feature Network	91.3%	78.8%	85.5%	85.2%	BraTS2020	[48]
MobileNetV2 with residual blocks as encoder and upsampling part of U-Net as decoder	91.4%	83.3%	88.1%	87.6%	BraTS2020	[28]
Asymmetric U-Net embedding network for 3D brain tumor segmentation	80.7%	69.7%	75.2%	75.2%	BraTS2020	[49]
Deep Convolutional Neural Networks with spherical space transformed input data	86.9%	79.0%	80.7%	82.2%	BraTS2020	[50]
Context Aware 3D UNet for Brain Tumor Segmentation	89.1%	79.1%	84.7%	84.3%	BraTS2020	[51]
Cascade of three Deep Layer Aggregation neural networks	88.6%	79.0%	83.0%	83.5%	BraTS2020	[52]
Multi-encoder Network for brain tumor segmentation	70.2%	73.9%	88.3%	77.5%	BraTS2020	[53]
3D-Znet encoder-decoder Network for 3D brain tumor segmentation	90.6%	84.5%	85.9%	87.0%	BraTS2020	Current

## Data Availability

All software, pretrained 3D-Znet models, and end-to-end electronic python note-book used in this study are available in the project GitHub repository (https://github.com/SOCR/DL_ZNet_3D_BrainSeg, accessed on 6 March 2023). The raw or compressed NIFTI files can also be easily displayed using the SOCR BrainViewer webapp (https://socr.umich.edu/HTML5/BrainViewer/, accessed on 6 March 2023).
